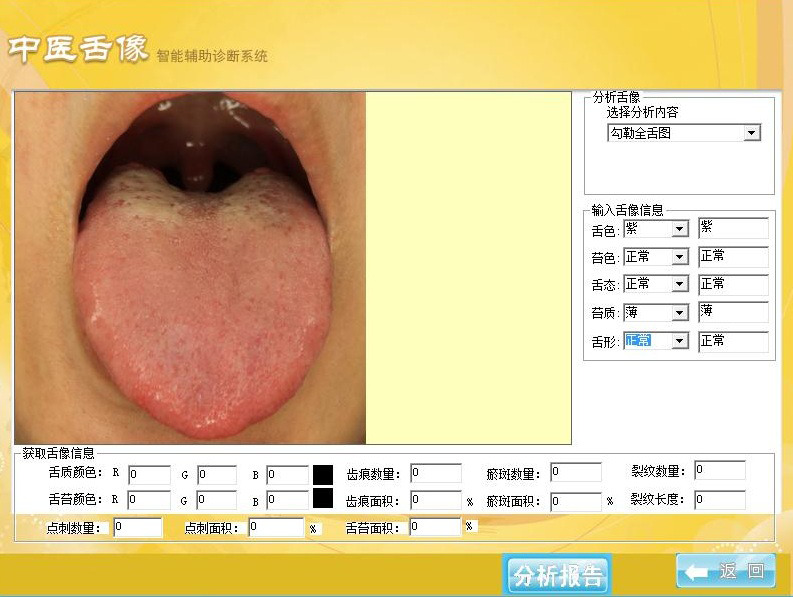# Association between color value of tongue and T2DM based on dose-response analyses using restricted cubic splines in China: A cross-sectional study

**DOI:** 10.1097/MD.0000000000038575

**Published:** 2024-06-21

**Authors:** Zhikui Tian, Xuan Sun, Dongjun Wang, Hongwu Wang

**Affiliations:** a School of Rehabilitation Medicine, Qilu Medical University, Zibo, China; b School of Health Sciences and Engineering, Tianjin University of Traditional Chinese Medicine, Tianjin, China; c College of Traditional Chinese Medicine, North China University of Science and Technology, Tangshan, China.

**Keywords:** dose–response relationship, tongue features, tongue image, traditional Chinese medicine, type 2 diabetes mellitus

## Abstract

This study aimed to explore the relationship between international commission on illumination (CIE) L*a*b* color value of tongue and type 2 diabetes mellitus (T2DM). We used restricted cubic spline method and logistic regression method to assess the relationship between CIE L*a*b* color value of tongue and T2DM. A total of 2439 participants (991 T2DM and 1448 healthy) were included. A questionnaire survey and tongue images obtained with tongue diagnosis analysis-1 were analyzed. As required, chi-square and *t* tests were applied to compare the T2DM and healthy categories. Our findings suggest the 95% confidence interval and odds ratio for body mass index, hypertension, and age were 0.670 (0.531–0.845), 13.461 (10.663–16.993), and 2.595 (2.324–2.897), respectively, when compared to the healthy group. A linear dose-response relationship with an inverse U-shape was determined between CIE L* and CIE a* values and T2DM (*P* < .001 for overall and *P* < .001 for nonlinear). Furthermore, U-shaped and linear dose-response associations were identified between T2DM and CIE b* values (*P* = .0160 for nonlinear). Additionally, in adults, the CIE L*a*b* color value had a correlation with T2DM. This novel perspective provides a multidimensional understanding of traditional Chinese medicine tongue color, elucidating the potential of CIE L*a*b* color values of tongue in the diagnosis of T2DM.

## 1. Introduction

The worldwide spread of type 2 diabetes mellitus (T2DM) can be attributed to a combination of environmental, genetic, and overnutrition-related aspects.^[1,2]^ An estimated 463 million adults between the ages of 20 and 79 have diabetes, representing an incidence of 9.3%. China is home to the most diabetic adults in the globe, comprising an estimated 116 million adults. Among this population, type 2 diabetes affects over 90% of the adults.^[3]^ As a chronic metabolic disorder, T2DM is depicted by resistance to insulin and hyperglycemia. Long-term poor blood sugar control can easily induce various complications such as heart, kidney, and nervous system.^[4]^ In addition, some studies have found a correlation between diabetes and some oral manifestations.^[5–7]^As early as 2000 years ago, the ancient Chinese medical book “Huangdi Neijing” has records of the diagnosis and treatment of diabetes.^[8]^ In traditional Chinese medicine (TCM), diabetes is called “Xiaoke” disease, which is caused by “Yin deficiency” and leads to frequent drinking and urination. Natural compounds such as curcumin and mangiferin are effective in treating diabetes complications.^[9]^ In clinical treatment, TCM has accumulated rich and detailed diagnostic experience and formed a systematic diagnostic standard and process.^[10,11]^ As the principal method by which TCM identifies Yin-Yang disorders,^[11]^ tongue diagnosis enables the early detection of numerous ailments. Individualized syndrome differentiation and treatment is an important characteristic of TCM.^[12]^ In recent years, tongue diagnosis has become an especially important noninvasive method for providing clinical data, particularly when diagnosing diabetes clinically; it also portrays a crucial function in diagnosing TCM. In contrast, with the ability to rapidly disclose a vast array of pathological data regarding the human body, tongue diagnosis is vital as a TCM diagnostic approach.^[13]^ However, the judgment of tongue diagnosis is considered easily influenced by different light sources and the subjective opinions of different doctors. In order to circumvent this drawback, we utilized an instrument for tongue diagnosis analysis-1 (TFDA-1) digital tongue diagnosis, which was produced by the Intelligent Diagnostic Technology Research Laboratory at Shanghai University of TCM, in order to gather objective purple tongue color values.^[14–18]^ A high-definition camera and standard light illumination are integrated into TFDA-1 to ensure a consistent light intensity and produce high-resolution tongue images suitable for post-analysis. Conversely, the instrument for tongue diagnosis was created with the intention of enhancing and standardizing TCM diagnostic procedures.^[15]^

In light of the progress made in TCM and big data technology, a multitude of research has been undertaken to examine tongue color attributes’ correlation with diabetes or metabolic syndrome through the utilization of big data. Jun Li et al used Vector Quantized Variational Autoencoder to extract the tongue picture features of diabetic patients, and used K-means to divide diabetic patients into 4 clusters, with highest classification accuracy is 87.8%.^[16]^ Yang Xiang et al diagnosed type 1 and type 2 diabetes by using a machine learning algorithm called random forest, and the accuracy of correct classification was 0.85.^[19]^ Shu-Jie Xia et al collected physiochemical and TCM indexes information from 450 patients with metabolic syndrome and selected the best-performing model by comparing the performances of 4 machine learning models, among which random forest had the best results and an accuracy of 0.942.^[20]^

These studies were all about classifying and diagnosing diabetes through methods such as machine learning. Although Jun Li et al’s included parameters such as L* value,^[16]^ to our knowledge, there are currently no studies on L*a*b* values in diabetic tongues. There is currently little evidence on the relationship between L*a*b* values and T2DM. After consideration of the unclear dose–response relationships, we conducted this study using objectively collected data from diabetic tongue to explore the associations between L*a*b* values and T2DM.

## 2. Materials & methods

### 2.1. Study population

A cross-sectional research was undertaken at the Second Affiliated Hospital of Tianjin University of TCM and Tianjin Academy of Integrative Medicine in Tianjin, China, encompassing a healthy group and a cohort with T2DM, from January 2019 to October 2020. The study comprised a total of 2439 subjects, 1448 of whom were asymptomatic and 991 who had T2DM. The following were the exclusion criteria: Must be <18 years old; Neglected to fulfill the requirements that the questionnaire provided; Individuals afflicted with mental disorders or other severe ailments; Women who are pregnant or lactating; Could not ensure cooperation with the image collector for the complete tongue; Incomplete clinical data; Patients with type 1 diabetes mellitus or diabetes-related acute complications; Patients with tumors, hematological system disorders, and other serious illnesses; Patients severely impacted by diet, drugs, or diseases. These patients agreed to fill out the questionnaires and take their tongue images using TFDA-1. In addition to receiving approval from the institution’s ethics committee, each subject provided informed consent prior to undergoing the biopsy (Supplementary Figure S2, Supplemental Digital Content, http://links.lww.com/MD/M922). This study was approved by the institutional ethics committee of Tianjin University of TCM, China (No: TJUTCM-EC20190004).

### 2.2. Data collection

The age, sex, and blood pressure of each participant were recorded. Body mass index (BMI) was divided into 2 groups: <24kg/m^2^ and ≥24kg/m^2,[21]^ age was divided into the 5 groups: <30, 30 to 39, 40 to 49, 50 to 59, >60. Throughout the duration of the research, patients were categorized as smokers if they consumed a minimum of 1 cigarette daily. The BMI was determined by quotient of the weight (in kg) and the square of the height (in m). Three minutes after the 10-minute seated period, the blood pressure of the nondominant arm was measured. The criteria for hypertension were diastolic blood pressure of at least 90 mm Hg or systolic blood pressure of at least 140 mm Hg.^[22]^ The proficient investigators affiliated with the School of Health Sciences and Engineering at Tianjin University of TCM talked to each subject with the help of a uniform questionnaire (Supplementary Figures S3, Supplemental Digital Content, http://links.lww.com/MD/M923).

#### 2.2.1. Tongue diagnostic instrument parameter settings

The Intelligent Diagnostic Technology Research Laboratory at Shanghai University of TCM developed the TFDA-1 desktop tongue diagnostic instrument for this study. The parameter settings were as follows: light source, cool white LED light; illuminance, 2354 lux; constant temperature of the colored light source, 5000 K, M mode; shutter speed, 1/125; aperture, F6.3; ISO sensitivity, 200.

#### 2.2.2. Requirements for tongue image acquisition

Refer to the tongue image collection process stipulated by the research group of the National Key Research and Development Program. First, the tongue diagnostic instrument’s parameters were checked, and the instrument’s outer edge and the internal contact parts with the patient were disinfected. The TFDA-1 tongue instrument was used to collect tongue images in the morning before breakfast. To prevent moss staining, the patient was asked to avoid strenuous activities, stay calm, reduce mood swings, and avoid drugs and foods that may cause moss staining 30 minutes before collection. If food residues did not affect the tongue’s or the lichen’s color, the patient was asked to rinse their mouth, and collection was initiated after 3 to 5 minutes. When shooting, the patient must sit upright, lean forward slightly, place their lower jaw against the instrument’s jaw support, close their eyes, and stretch out their tongue after turning on the light source such that the tongue was relaxed and centered, the tongue surface was flat, and the tip of the tongue was downward, eventually fully exposing the tongue with 1/2 to 2/3 of the tongue protruding. Then, the middle of the tongue was tapped on the screen to take a photo. After shooting, the photos were removed, and anomalies such as excessive exposure, insufficient light, and fog occlusion were eliminated.

### 2.3. Diagnostic criteria

A prior diagnosis of T2DM at the Second Affiliated Hospital of Tianjin University of TCM, fasting plasma glucose of at least 7.0 mmol/L, or 2h plasma glucose of at least 11.1 mmol/L, was sufficient to establish the condition.^[23]^ In healthy group, participants’ blood glucose did not meet the criteria above, and could not have health problems or medical history records. Healthy status was defined as the absence of acute or chronic illness and good mental health, which allowed for fluent participation in the question-and-answer process. Fatigue and long-term insomnia were the most common mental illnesses; chronic diseases included hypertension and glucose-lipid metabolism disorders, persistent benign or malignant diseases that could interfere with study objectives, and the absence of oral disease.

### 2.4. CIE-Lab color value measurements

**Figure 1.**
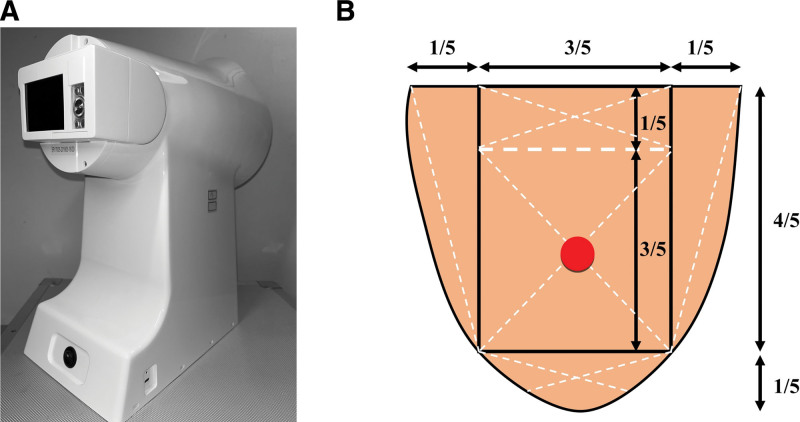
(A) TFDA-1 digital tongue diagnosis instrument. (B) The specifying spot on the tongue contour. TFDA-1 = tongue diagnosis analysis-1.

The TFDA-1 digital tongue diagnosis instrument, which was devised by the Intelligent Diagnostic Technology Research Laboratory at Shanghai University of TCM, was utilized to gather data on tongue characteristics from the subjects. The tongue color was determined by the parameters of the TFDA-1 device, we converted the color values from RGB to international commission on illumination (CIE) L*a*b* value, indicating the values L*, a*, and b*, where CIE L* value represented the lightness from 0 to 100 (black to white axis) and CIE a* value and CIE b* value represented the chromaticity, where CIE a* value was the measurement from127 to −128 (red to green axis) and CIE b* value was the measurement from127 to −128 (yellow to blue axis). The CIE L*, a*, and b* values by specifying spots on the tongue contour are shown in Figure 1. Additionally, the color change was calculated using the following formulas:

$$\begin{array}{l} \left[\begin{array}{l} X\;Y\;Z\; \end{array}\right]=\left[\begin{array}{lllllll} 0.412453 & 0.357580 & 0.180423\;0.212671 & 0.715160 & 0.072169\;0.019334 & 0.119193 & 0.950227\; \end{array}\right]\left[\begin{array}{l} R\;G\;B\; \end{array}\right] \end{array}$$ (1)

Then, the transformation from CIEXYZ color space to CIE-LAB color space is done by the following formulas:

$$\begin{array}{l} \begin{array}{llll} & L^{*}=116f\left(Y/Y_{n}\right)-16\; & a^{*}=500\left[\;f\left(X/X_{n}\right)-f\left(Y/Y_{n}\right)\right]\; & b^{*}=200\left[\;f\left(Y/Y_{n}\right)-f\left(Z/Z_{n}\right)\right]\; \end{array} \end{array}$$ (2)

$$\begin{array}{l} f(t)=\left\{ \begin{array}{lll} t^{1/3} & \;if\;t>{\left(\frac{6}{29}\right)}^{3}\;\frac{1}{3}{\left(\frac{29}{6}\right)}^{2}t+\frac{4}{29} & \;otherwise\;\; \end{array}\right. \end{array}$$ (3)

### 2.5. Statistical analysis

Utilizing R (version 3.6.3, http://www.r-project.org/), the statistical analysis was conducted; means ± SD for continuous variables exhibiting normal or approximate normality were provided. The median was utilized to characterize non-normal distributions (upper and lower quartiles). The presentation of categorical variables consisted of percentages and frequencies. As applicable, nonparametric tests, Student *t* tests for continuous parametric variables, and chi-squared tests for categorical ones were used to examine differences between groups. As stated, every level of statistical significance is 2-sided and has a 0.05 significance threshold. Utilizing univariate binary logistic regression, the relationship between variables and T2DM was determined. Subsequent to a univariate test for significance (*P* < .2), variables were chosen for multivariate analysis. The significance of the variables statistics-wise was considered when selecting them for the multivariate model. Restricted cubic splines (RCS) were applied to determine the potential nonlinear relationship between CIE L*a*b* values and T2DM.

## 3. Results

### 3.1. Patient characteristics

There were 2439 subjects in this investigation; 991 had type 2 diabetes and 1448 were in good health, 483 male participants (48.7%) had T2DM and 508 female participants (51.3%) had T2DM. When compared with the healthy group, the participants with T2DM were older (age > 60, 8.0% vs 3.7%) and overweight (BMI ≥ 24, 59.1% vs 53.0%). The proportions of hypertension in the T2DM group were greater than healthy group (Table 1).

**Table 1:** Characteristics of participants (n = 2439).

Characteristics	Healthy (n = 1448)	T2DM (n = 991)	*χ*^2^/*t*/*Z*	*P* value
Age (yr), n (%)			716.467	.001
<30	323 (22.3)	14 (1.4)		
30–39	594 (41)	124 (12.5)		
40–49	296 (20.4)	238 (24)		
50–59	182 (12.6)	536 (54.1)		
>60	53 (3.7)	79 (8.0)		
Smoking, n (%)			2.216	.137
None	1105 (76.3)	730 (73.7)		
Yes	343 (23.7)	261 (26.3)		
Hypertension, n (%)			891.168	.001
Yes	1260 (87)	273 (27.5)		
BMI (kg/m^2^), n (%)	188 (13)	718 (72.5)	8.846	.003
<24	680 (47)	405 (40.9)		
≥24	768 (53)	586 (59.1)		
Gender, n (%)			0.142	.706
Male	717 (49.5)	483 (48.7)		
Female	731 (50.5)	508 (51.3)		

BMI = body mass index, T2DM = type 2 diabetes mellitus.

**Figure 2.**
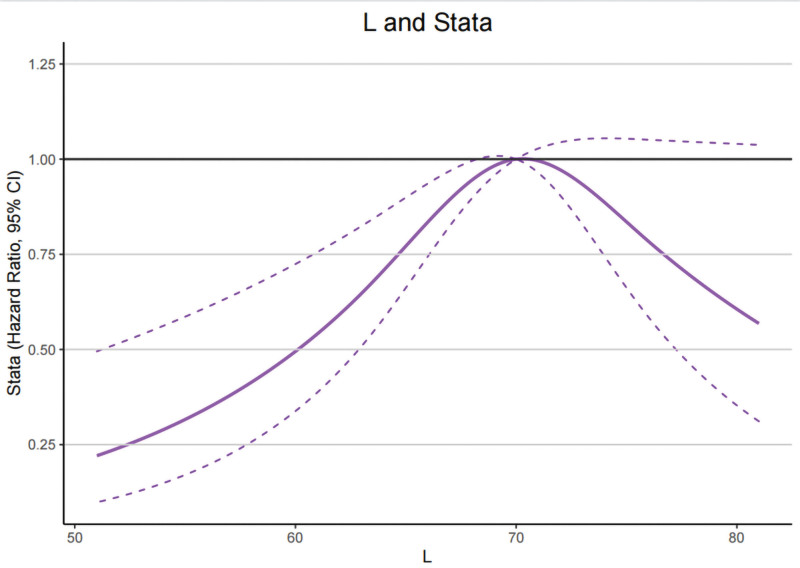
Restricted cubic spline models of the odds ratios (ORs) of T2DM with CIE L* values. The solid line and dashed lines represent the estimated ORs and the 95% confidence intervals, respectively. CIE = international commission on illumination, T2DM = type 2 diabetes mellitus.

Photos of tongue color delivery were processed using the software “TCM Tongue Image Intelligent Auxiliary Diagnosis System” for determination of color parameters in the CIE-Lab system also (Supplementary Figures S1, Supplemental Digital Content, http://links.lww.com/MD/M921). In Figure 2, We measured tongue color parameters at an equal point under the same lighting conditions. As can be seen from Table 2, the tough color differs from the 2 groups, namely: The CIE a* values of T2DM were significantly higher than healthy (*P* = .001). Moreover, in the 2 groups, there was a substantial difference in the CIE b* values (*P* = .013).

**Table 2:** Tongue color parameters (n = 2439).

Characteristics	Healthy (n = 1448)	T2DM (n = 991)	*χ*^2^/*t*/*Z*	*P* value
L	69.52 ± 6.53	69.85 ± 4.54	−1.413	.158
a	14.19 ± 4.28	15.40 ± 3.76	−7.245	.001
b (median; IQR)	4.00 (2.00–5.00)	4.00 (2.00–5.00)	−2.474	.013

IQR = interquartile range, T2DM = type 2 diabetes mellitus.

### 3.2. Association between CIE L*a*b* values of tongue and T2DM

The models were fitted with univariate and multivariate logistic regression analyses in order to ascertain the attributes that are correlated with T2DM. Table 3 shows the association between CIE L*a*b* tongue values and T2DM. The analysis using univariate logistic regression unveiled that variables such as age, smoking, hypertension, BMI, and CIE a* value. In the multivariate-adjusted model, age, hypertension, BMI, and CIE L*a*b* values were defined as covariates (Table 4), the results indicate that elderly people and hypertension were associated with T2DM. L*a*b* values were associated with T2DM. The odds ratios (ORs) for CIE L*a*b* values were 1.017 (0.992–1.043), 1.068 (1.035–1.102), and 0.989 (0.935–1.046), respectively. Notably, after adjusting for age, the results demonstrated that CIE L* and CIE a* values remained significantly positively associated with T2DM. The ORs for L* and a* values were 1.027 (1.002–1.053) and 1.078 (1.046–1.112), respectively.

**Table 3:** The associations between CIE L*a*b* tongue values and T2DM (n = 2439).

Parameters	*B*	SE	OR	95% CI	*P* value
Age					
30–39	1.466	0.310	4.334	2.361–7.955	.001
40–49	2.491	0.308	12.069	6.604–22.056	.001
50–59	3.644	0.307	38.238	20.958–69.767	.001
>60	3.253	0.364	25.871	12.673–52.815	.001
Smoking					
Yes	0.788	0.136	2.199	1.684–2.870	.001
Hypertension					
Yes	2.765	0.129	15.883	12.341–20.443	.001
BMI					
≥24	−0.521	0.123	0.594	0.466–0.756	.001
L	0.017	0.013	1.017	0.992–1.043	.193
a	0.066	0.016	1.068	1.035–1.102	.001
b	−0.011	0.028	0.989	0.935–1.046	.703

BMI = body mass index, CI = confidence interval, CIE = international commission on illumination, OR = odds ratio, T2DM = type 2 diabetes mellitus

**Table 4:** Multivariate logistic model of T2DM across age-adjusted (n = 2439).

Parameters	*B*	SE	OR	95% CI	*P* value
Age	0.954	0.056	2.595	2.324–2.897	.001
Hypertension					
Yes	2.600	0.119	13.461	10.663–16.993	.001
BMI					
≥24	−0.401	0.119	0.670	0.531–0.845	.001
L	0.027	0.013	1.027	1.002–1.053	.033
a	0.076	0.016	1.078	1.046–1.112	.001
b	−0.019	0.028	0.981	0.928–1.036	.490

BMI = body mass index, CI = confidence interval, OR = odds ratio, T2DM = type 2 diabetes mellitus.

**Figure 3.**
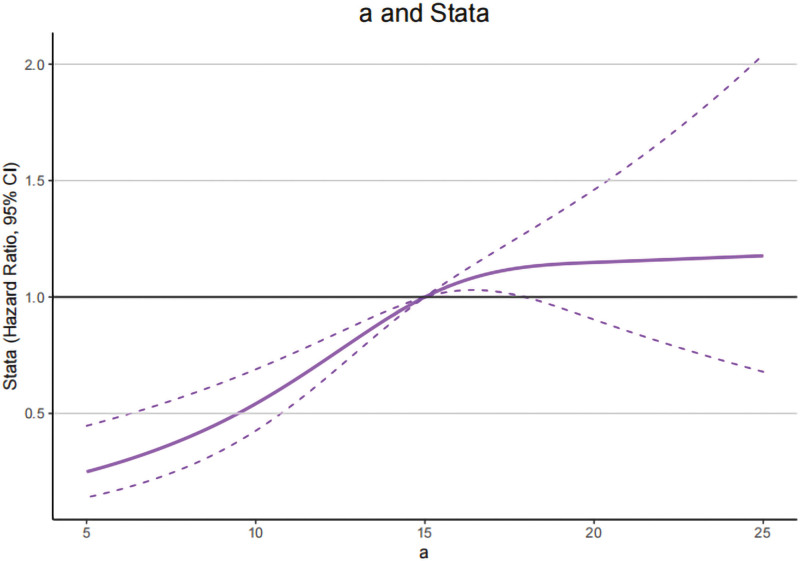
Restricted cubic spline models of the odds ratios (ORs) of T2DM with CIE a* values. The solid line and dashed lines represent the estimated ORs and the 95% confidence intervals, respectively. CIE = international commission on illumination, T2DM = type 2 diabetes mellitus.

**Figure 4.**
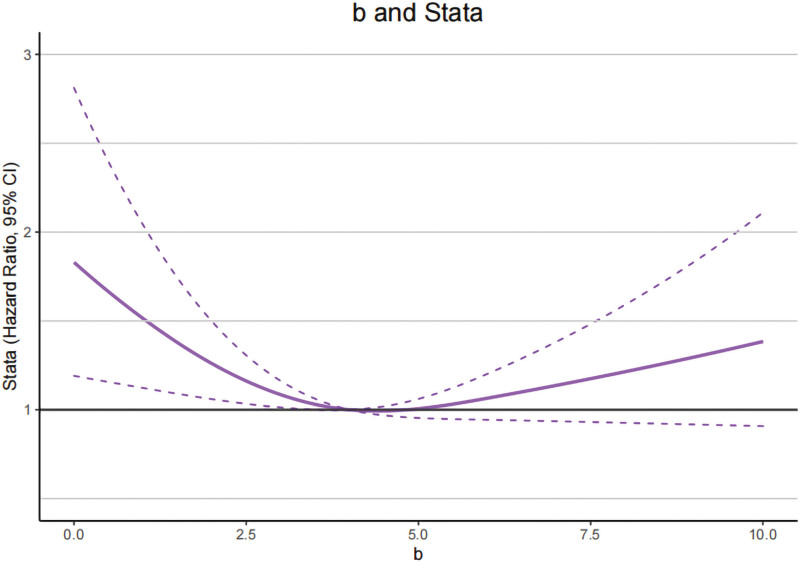
Restricted cubic spline models of the odds ratios (ORs) of T2DM with CIE b* values. The solid line and dashed lines represent the estimated ORs and the 95% confidence intervals, respectively. CIE = international commission on illumination, T2DM = type 2 diabetes mellitus.

To further explore the potential relationship between CIE L*a*b* values of tongue and T2DM. In RCS model, we analyzed the distribution and the changing trend of OR about CIE L*a*b* values for T2DM and then discovered a significant inverse U-shaped relationship between CIE L* and CIE a* values in T2DM (*P* for overall < .001, *P* for nonlinear < .001), while this relationship was statistically significant for CIE b* value (*P* for nonlinear = .0160), coming out that the curve presents a trend to ascend until the CIE L* and CIE a* value of around 68.2 and 15.0 and then descend, whereas for CIE b*value this trend is to descend first and then ascend, this interception were 3.5 (Figs. 2–4).

## 4. Discussion

Color of tongue has been used as a qualitative indicator for diagnosis of the disease in TCM.^[24–27]^ Some research compared the value of CIE L*a*b* of tongue colors and demonstrated that the classification of the objective tongue is feasible in TCM.^[28,29]^ Duan et al also found that b values in the Lab color space differed between patients with esophageal cancer and normal subjects.^[30]^ Similar results showed differences in Lab values of the tongue and coating for lung cancer patients with 4 syndrome groups.^[31]^ Lower CIE a* and higher CIE b* values of yellow tongue coating in patients with metabolic-associated fatty liver disease are associated with carbohydrate metabolism ailments and oral microbiome inflammation, according to a previous study on the oral microbiome.^[32]^ Hyperglycemia and chronic diabetes both elevate the likelihood of developing microvascular or macrovascular complications. In addition, the majority of diabetic patients presented with oral indications including candidiasis, lichen planus, geographic tongue, loss of tooth, xerostomia, periodontal disease, caries, scorching mouth disorder, dysfunction of taste and the salivary gland, and delayed wound healing. Additionally, buccal alterations may be found in diabetic individuals. A potential explanation for the relationship between diabetes and the tongue may be that diabetes and high blood sugar levels cause microvascular or macrovascular complications.^[33,34]^ Moreover, most diabetic patients have different oral manifestations, and these clinical manifestations will have different effects on the color of the tongue.^[35]^

Research conducted by Po-Chi Hsu et al indicated that patients with T2DM (199 T2DM, and 372 non-DM individuals as control) area of yellow fur, thick fur, and bluish tongue are significantly larger than non- diabetes individuals. Thick fur, yellow fur color, and bluish tongue were more common in T2DM, and tongue diagnosis could be used as a simple, noninvasive tool for pre-diagnosing diabetes.^[36]^ By determining the CIE-LAB color parameters, the influence of tongue color on the color of T2DM was investigated.

Significant variations were identified in the correlation between L*a*b* tongue values and the T2DM. In regard to the objective assessment of the diabetic tongue, 2 color parameters that are highly sensitive to color assessment in the mainly red tongue appear to be crucial. The CIE a* value identifies whether an object is red (positive a*) or green (negative a*). The mean CIE a* value for individuals with T2DM was 15.4, surpassing the value of 14.19 observed in the control group (Table 2).

A higher CIE a* value in diabetic tongue could be attributed to dilation of blood vessels due to heat. According to TCM theory, redder tongue mirrors an interior heat pattern.^[37,38]^ Therefore, the color of tongue, which is based on T2DM and normal group, probably reflects the redder colors of the T2DM. The other important color parameter in diabetic tongue is CIE b* value, which indicates the yellow (positive b*) and blue (negative b*). The U-shaped dose-response association between CIE b* value and T2DM was identified in this current study, parallel with the findings of Tomooka K et al.^[39]^ A higher CIE b* value in diabetic tongue could be attributed to thicker fur on the tongue.

TCM theory posits a connection between tongue fiber and the digestive system. Typically, a yellow tongue is supplemented by blood stasis and dampness of phlegm.^[40]^ In a related study of yellow tongues in diabetics, Po-Chi Hsu et al indicated that yellow fur appeared significantly more frequently in patients with T2DM than in nondiabetic groups.^[36]^ Another study on the incidence of diabetes showed a higher prevalence in the presence of yellow tongue moss, which was also associated with prediabetes.^[39]^ As an indicator of the degree of tongue lightness in diabetics, the CIE L* value was greater in magnitude compared to the control group. Hence, attributes that thicken the tongue appear to affect its lightness in diabetics. There was a correlation between greasy and denser coatings and oxygen-free radical damage.^[41,42]^ In TCM theory, as the disease slowly develops, the thin white tongue begins to gradually whiten and thicken. Therefore, the degree of thick tongue was associated with T2DM.

According to some studies, machine learning technology was used to create a diabetes risk prediction model by extracting tongue features such as color values and texture features. The model was then screened and tested for performance. This model was primarily used for the early detection of diabetes.^[11,14,43]^ Given the difficulty distinguishing tongue color, Zhang et al developed a multicolor feature fusion method to improve diagnostic results.^[44]^ However, there is still a lack of research on the objectification value of tongue color in patients with T2DM. This study explored the variables that might affect T2DM through a nonparametric fitting method: limited cubic spline. Compared to the machine learning method, the limited cubic spline has higher flexibility by using an appropriate number of nodes to fit the shape of the curve and explore the nonlinear relationship between the objectified value of tongue color and type 2 diabetes. The threshold of this objectified value in the results can provide a reference for researching tongue color in TCM and can assist in clinical diagnosis.

Previously, it was suggested that a color measurement was basically made up of a single point in space, so it is logical to take 3 coordinates.^[45]^ Under a disease condition, however, such as in this study of clinical diagnosis of tongue in diabetic patients, it would be advantageous to use only 1 (CIE a* value) or 2 (CIE a* value and CIE b* value) variables rather than 3. Because the CIE a* value is highly similar to the color of the tongue and is easier to measure, CIE a* values alone could be used as a quick and objective color score in diabetic tongue diagnosis.

Although this study explored the relationship between CIE L*a*b* values and T2DM, there were still some limitations. First, for the first time, RCS was used to analyze CIE L* a* b* values in the T2DM tongue. Second, we considered and controlled age factors. However, some limitations should be taken into our results. Due to the fact that the populations were sourced from a solitary center located in Tianjin City, the generalizability of these results to different cities or countries is uncertain. Second, a cross-sectional study could not make any causal inferences. Third, some related factors of T2DM such as low-density lipoprotein-C, and glycosylated hemoglobin, type A1C were not included in this study. Fourth, our questionnaire did not collect information on socioeconomic data, which may have a potential effect on our results. Finally, we could not rule out the effect of participants’ diet or medications on diabetic tongue.

## 5. Conclusions

TCM believed that T2DM could affect the tongue’s color, and several related studies confirmed this. However, there was a lack of research to objectively evaluate the relationship between tongue color and type 2 diabetes. According to our understanding, this is the first study to use objective data to investigate the associations between T2DM in Chinese adults and CIE L*a*b* values of the tongue. In this cross-sectional study, we looked at adults with T2DM and those who were healthy in Tianjin, China. Following logistic regression analysis, the results show that CIE a* values of the tongue increased in T2DM patients. Additionally, elevated CIE L* and a* tongue levels were linked to T2DM after controlling for demographics. We also used RCS to investigate the relationship between CIE L*a*b* values of the tongue and T2DM. The tongue’s CIE L* and CIE a* values showed an inverse U-shaped association with T2DM. Further objectification of tongue color may provide new clues for type 2 diabetes intervention and allow for assessing T2DM status based on changes in tongue color. Meanwhile, the study discovered that overweight and hypertensive people have a higher prevalence of T2DM and that T2DM remission can be achieved through intensive lifestyle changes, emphasizing the importance of identifying effective strategies to promote lifestyle changes in this population. Simultaneously, controlling influencing factors, researching new bioinformatics analysis methods related to TCM, and conducting animal experiments will help prevent and treat type 2 diabetes in the future. Our next research focus will be collecting TCM “bianzheng” differentiation from different T2DM patients to determine if the objective CIE L*a*b* color values differ in different “bianzheng” differentiation states and determine its threshold.

## Acknowledgments

The authors appreciate the National Key Research and Development Program of China for the grant support.

## Author contributions

**Conceptualization:** Zhikui Tian, Hongwu Wang.

**Data curation:** Zhikui Tian, Xuan Sun, Hongwu Wang.

**Formal analysis:** Zhikui Tian, Xuan Sun, Hongwu Wang.

**Funding acquisition:** Hongwu Wang.

**Investigation:** Dongjun Wang.

**Methodology:** Zhikui Tian, Xuan Sun.

**Software:** Dongjun Wang.

**Supervision:** Zhikui Tian, Dongjun Wang, Hongwu Wang.

**Validation:** Zhikui Tian, Dongjun Wang, Hongwu Wang.

**Visualization:** Zhikui Tian.

## Supplementary Material

**Figure:**
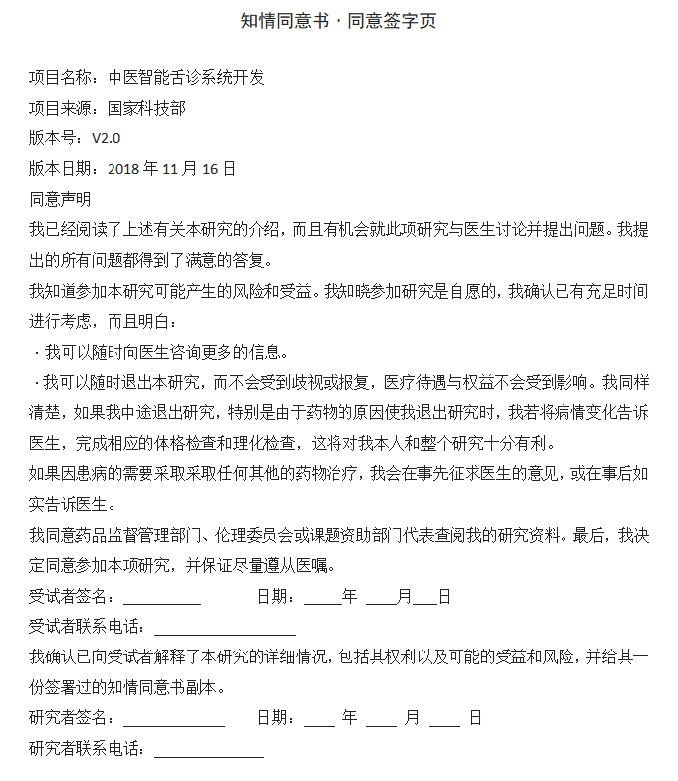


**Figure:**
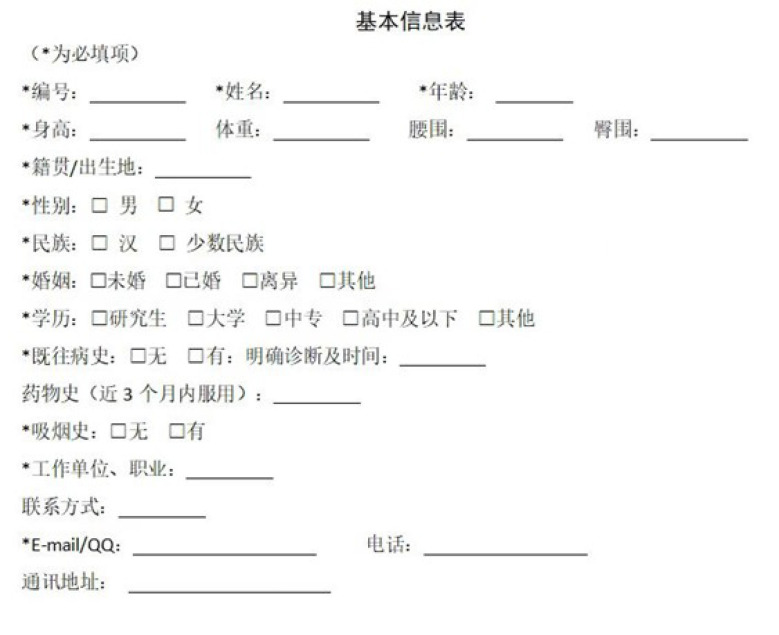


**Figure:**